# Genome sequence and genetic transformation of a widely distributed and cultivated poplar

**DOI:** 10.1111/pbi.12989

**Published:** 2018-10-24

**Authors:** Jianchao Ma, Dongshi Wan, Bingbing Duan, Xiaotao Bai, Qiuxian Bai, Ningning Chen, Tao Ma

**Affiliations:** ^1^ State Key Laboratory of Grassland Agro‐Ecosystem Institute of Innovation Ecology & School of Life Sciences Lanzhou University Lanzhou China; ^2^ Key Laboratory of Bio‐Resource and Eco‐Environment of Ministry of Education College of Life Sciences Sichuan University Chengdu China

**Keywords:** *Populus alba*, *Populus bolleana*, comparative genomics, transformation efficiency, gene families

## Abstract

*Populus alba* is widely distributed and cultivated in Europe and Asia. This species has been used for diverse studies. In this study, we assembled a *de novo* genome sequence of *P. alba* var. *pyramidalis* (= *P. bolleana*) and confirmed its high transformation efficiency and short transformation time by experiments. Through a process of hybrid genome assembly, a total of 464 M of the genome was assembled. Annotation analyses predicted 37 901 protein‐coding genes. This genome is highly collinear to that of *P. trichocarpa,* with most genes having orthologs in the two species. We found a marked expansion of gene families related to histone and the hormone auxin but loss of disease resistance genes in *P. alba* if compared with the closely related *P. trichocarpa*. The genome sequence presented here represents a valuable resource for further molecular functional analyses of this species as a new tree model, poplar breeding practices and comparative genomic analyses across different poplars.

## Introduction

Poplars have been selected as the model for a range of studies on trees at the molecular level for three reasons (Brunner *et al*., 2004). First, they were reported to be genetically transformable three decades ago (Fillatti *et al*., 1987). Second, poplars have a small genome size, short rotation cycle, easy *in‐vitro* regeneration and rapid vegetative propagation compared with other trees (Bradshaw *et al*., 2000; Brunner *et al*., 2004). Third, the genome sequence of one poplar species, *P. trichocarpa*, was reported more than a decade ago (Tuskan *et al*., 2006). A genetic transformation system for *P. trichocarpa* was established just after its genome had been reported (Song *et al*., 2006), it is still difficult to transform and grow this species in some labs or regions of the North Hemisphere. In numerous molecular studies on poplar have therefore used the *P. trichocarpa* genome for gene sequence and expression analyses, but for physiological and phenotypic tests other hybrid poplars have been transformed, for example, *P. tremula *× *tremuloides* (Ohtani *et al*., 2011), *P. alba* × *grandidentata* (Maloney and Mansfield, 2010), *P. alba* × *P. tremula* (Cho *et al*., 2016) and *P. simonii* × *P. nigra* (Zhao *et al*., 2017). Attributed to the different genetic backgrounds and gene sequences and/or variations in copy number of homologs between different species, such heterogeneous transformation (Han *et al*., 1997, 2000; Ma *et al*., 2004) may give rise to numerous unexpected results in phenotypic and molecular analyses. Therefore, it is necessary to sequence the genomes of more species, especially those with widespread distribution and cultivation. These genome resources are useful not only for functional dissection of genes and the genetic optimization of fibre and biomass production and abiotic stress resistance traits in these poplars, but also important for comparative genomic studies across different poplars.

*Populus alba*, called as the white poplar, is an ecologically and economically important species of the section *Populus* (Eckenwalder, 1996). This species is widely distributed and cultivated in Europe and Asia (Lazowski, 1997). The natural populations of this species hybridize frequently with other closely related species (for example, *P. tremula*) producing numerous natural hybrids (Lexer *et al*., 2005; Van Loo *et al*., 2008). This species has been widely used in the numerous labs for diverse studies (e.g. Lexer *et al*., 2005; Van Loo *et al*., 2008; Wang *et al*., 2008). The previous studies suggest that *P. alba* is easily genetically transformed (Soliman *et al*., 2017; Wang *et al*., 2008) and one genotype of this species can start to flower very quickly within only 9 months after being regenerated (Meilan *et al*., 2004). One variety of this species, var. *pyramidalis* (= *P. bolleana*) has been widely cultivated for urban afforestation, ecological restoration and wood use from northwest (Xinjiang) to northern China (Beijing) because of its rapid growth, lack of seed catkins, erect stems and high biomass production (Xu, 1988; Xu *et al*., 2011; Zhang *et al*., 2008). This variety was selected, domesticated and clonally propagated by means of branch cuttings from one or a very limited number of male individuals of *P. alba* obtained from its native, dryland distributions in central Asia (Yang *et al*., 1992). The cutting clones of var. *pyramidalis* usually start to flower within 5 years. In this study, we firstly sequenced the genome of this variety and compared the genomic differences between it and the closely related species. We then confirmed the high transformation efficiency of *P. alba* as reported before (Soliman *et al*., 2017; Wang *et al*., 2008). We believe that this genome resource will be highly useful for molecular analyses of the gene functions in poplar trees and comparative genomic analyses across different poplars.

## Results

### Genome sequencing, assembly and annotation

**Figure 1:**
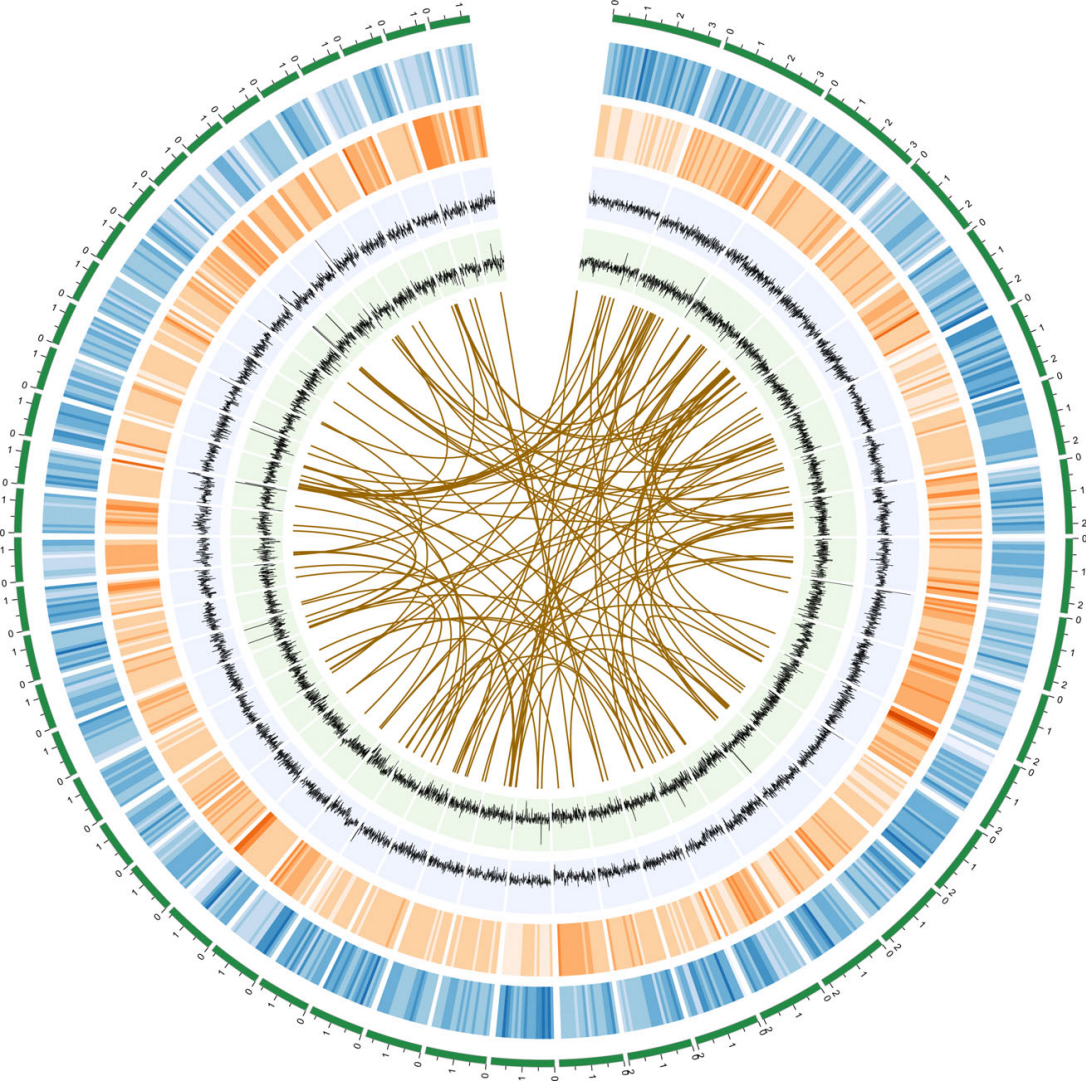
*Populus alba* var. *pyramidalis* genome. From the outer edge inward, circles represent the 50 largest DNA sequence scaffolds (green), the genes on each scaffold (blue), repeat density at 10 kb intervals (orange), GC density at 10 kb intervals (green), and the sequenced reads density at 10 kb intervals (grey). Links in the core connect duplicated sets of genes (*E*‐value threshold of <1e^−10^ and 85% similarity).

We sequenced the genome of a clonally propagated male individual of *P. alba* var. *pyramidalis* using a whole‐genome shotgun strategy. About 320× Illumina data were generated (Table S1) and assembled into an initial genome sequences spanning 406.8 Mb, with a contig N50 of 9.8 kb and a scaffold N50 of 348.9 kb (Figure S1A; Table S2). To overcome challenges posed by the relatively high number of repeats and heterozygosity of this genome (Figure S1B), we also generated about 30× PacBio RS raw data to improve this short‐read assembly. The size of the final assembly after removing scaffolds <1 kb in length comprised 17 797 scaffolds with contig and scaffold N50 size of 26 535 bp and 459 178 bp, respectively (Table S2), representing over 87% of the total genome size as estimated from *k*‐mer analysis (536 Mb) conducted using KmerGene software (Chikhi and Medvedev, 2013). Our assessment of the quality of the assembly suggested that most of the genome was assembled (Figure 1). A total of 201 Mb (44.61% of the genome) was annotated as consisting of repetitive sequences, similar to the values determined for genomes of other poplar species (Ma *et al*., 2013; Tuskan *et al*., 2006; Yang *et al*., 2017; Table S3). The heterozygosity level of *P. alba* var. *pyramidalis* was estimated to be 0.53% on the basis of mapping short library reads to the draft genome (2 394 196 SNPs and 414 130 indels).

A combination of *de novo* and homology‐based gene prediction generated a final gene setincluding 37 901 protein‐coding genes (Figure 1; Table S4), with the gene structures being refined using alignments with transcriptomes from four different tissue types (leaf, phloem, xylem and root; Table S5). Of these genes, 4779 were predicted to generate multiple transcript variants due to alternative splicing. The predicted genes were then functionally annotated by a consensus approach, using InterPro (Hunter *et al*., 2008), Gene Ontology (GO; Ashburner *et al*., 2000), Kyoto Encyclopedia of Genes and Genomes (KEGG; Kanehisa and Goto, 2000) and Swiss‐Prot (Boeckmann *et al*., 2003). In total, 32 513 genes (85.8% of the predicted genes) have known homologs in protein databases (Table S6). We further assessed the completeness of the genome assembly, based on comparison with a benchmark of 429 conserved eukaryote genes using the benchmarking sets of universal single‐copy ortholog (BUSCO) v3 method (Simão *et al*., 2015). The results indicated that our annotation of the *P. alba* var. *pyramidalis* genome is nearly complete, with 91.10% of the complete BUSCOs, a value similar to *P. trichocarpa* and *P. euphratica* (Table S7). In addition, we also identified 569 ribosomal RNA (rRNA), 940 transfer RNA, 123 small nuclear RNA and 1050 microRNA genes in the assembled genome (Table S8).

### Comparative genome analysis

**Figure 2:**
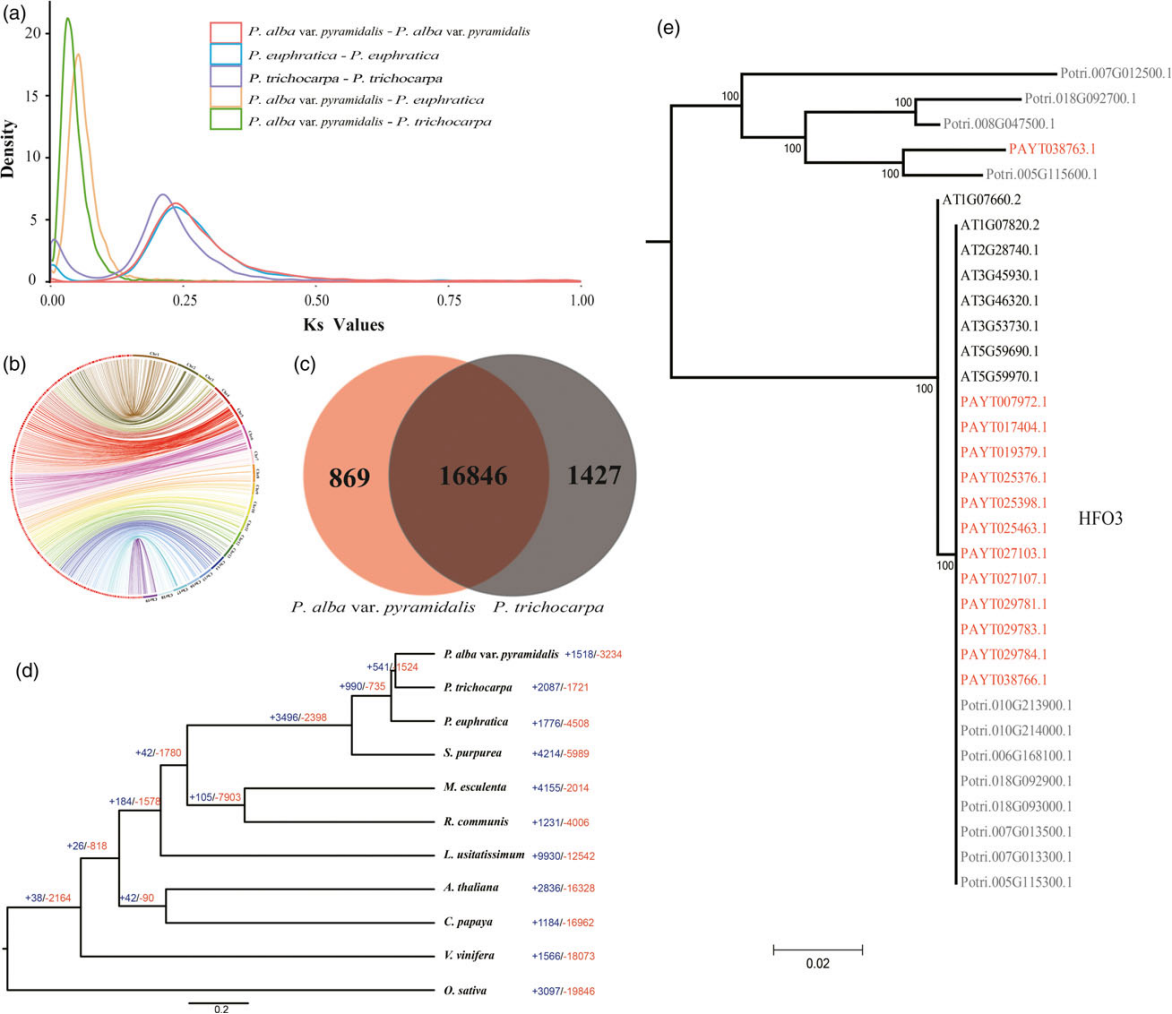
Genome characteristics. (a) Distribution of synonymous nucleotide substitutions (Ks). (b) Circos plots showing synteny between the *Populus alba* var. *pyramidalis* (left) and *P. trichocarpa* (right) genomes. (c) Venn diagram showing the number of gene families shared between *P. alba* var. *pyramidalis* and *P. trichocarpa*. (d) Expansion and contraction of gene families in ten species. (e) Phylogenetic trees of *HFO3* genes in *Arabidopsis*,* P. alba* var. *pyramidalis* and *P. trichocarpa*.

Phylogenetic analysis based on the genomic evidence suggested that *P. alba* var. *pyramidalis* is more closely related to *P. trichocarpa* than to *P. euphratica*. The divergence between *P. alba* var. *pyramidalis* and *P. trichocarpa* was estimated to have occurred ~13 Mya (Figure S1C). As expected, *P. alba* var. *pyramidalis* had the same whole genome duplications (WGD) as *P. trichocarpa* and *P. euphratica* (Figure 2A). In addition, we identified a total of 3363 collinear blocks of about 300 Mb in length between *P. alba* var. *pyramidalis* and *P. trichocarpa* (Figure 2B). *P. alba* var. *pyramidalis* shared 16 846 gene families (including 28 710 genes) with *P. trichocarpa*, representing 76% of the total annotated genes (Figure 2C; Figure S1D). We further performed tests for deviations in the Ka/Ks ratio (non‐synonymous substitutions per non‐synonymous site to synonymous substitutions per synonymous site) for these homologous genes and 865 gene pairs were identified to have high diversification ratios (Ka/Ks > 1). GO enrichment indicated these genes were mainly functioned in “primary metabolic process” and “defense response”, including these well‐known defense response genes CPR1 (Kim *et al*., 2010), LEA (Salleh *et al*., 2012), and BIR1 (Zhang *et al*., 2013; Table S9). Besides, we found that there were 869 *P. alba* var. *pyramidalis* specific gene families (Figure 2C), which were also enriched in ‘defense response’ (GO:0006952, 76 genes, *P *=* *2.17 × 10^−20^), including eight families containing ‘salt stress response/antifungal’ domains. Whereas 1427 gene families specific to *P. trichocarpa* were enriched in ‘photosystem II reaction center’ (GO:0009539, 11 genes, *P *=* *2.67 × 10^−13^; Figure S2).

### Gene family expansion and contraction

We found that 1518 gene families were expanded in the *P. alba* var. *pyramidalis* genome compared to other plant species (Figure 2d). GO enrichment showed that these expanded families were significantly enriched in the terms ‘ADP binding’ (GO:0043531, 103 genes, *P *=* *2.06 × 10^−14^), ‘defense response’ (GO:0006952, 193 genes, *P *=* *5.47 × 10^−12^), and ‘secondary metabolic process’ (GO:0019748, 174 genes, *P *=* *1.35 × 10^−9^; Table S10). Among these families, PUP and auxin/indole‐3‐acetic acid (Aux/IAA) genes related to cytokinin and auxin responses were expanded with a high expression in phloem and xylem (Figure S3), probably related to the fast growth of this variety. In addition, we found that homologs of *Arabidopsis* HFO3 (Tenea *et al*., 2009) histone genes, which could increase *Agrobacterium*‐mediated transformation when over‐expressed (Tenea *et al*., 2009), were noticeably expanded in *P. alba* var. *pyramidalis* (Figure 2E).

**Figure 3:**
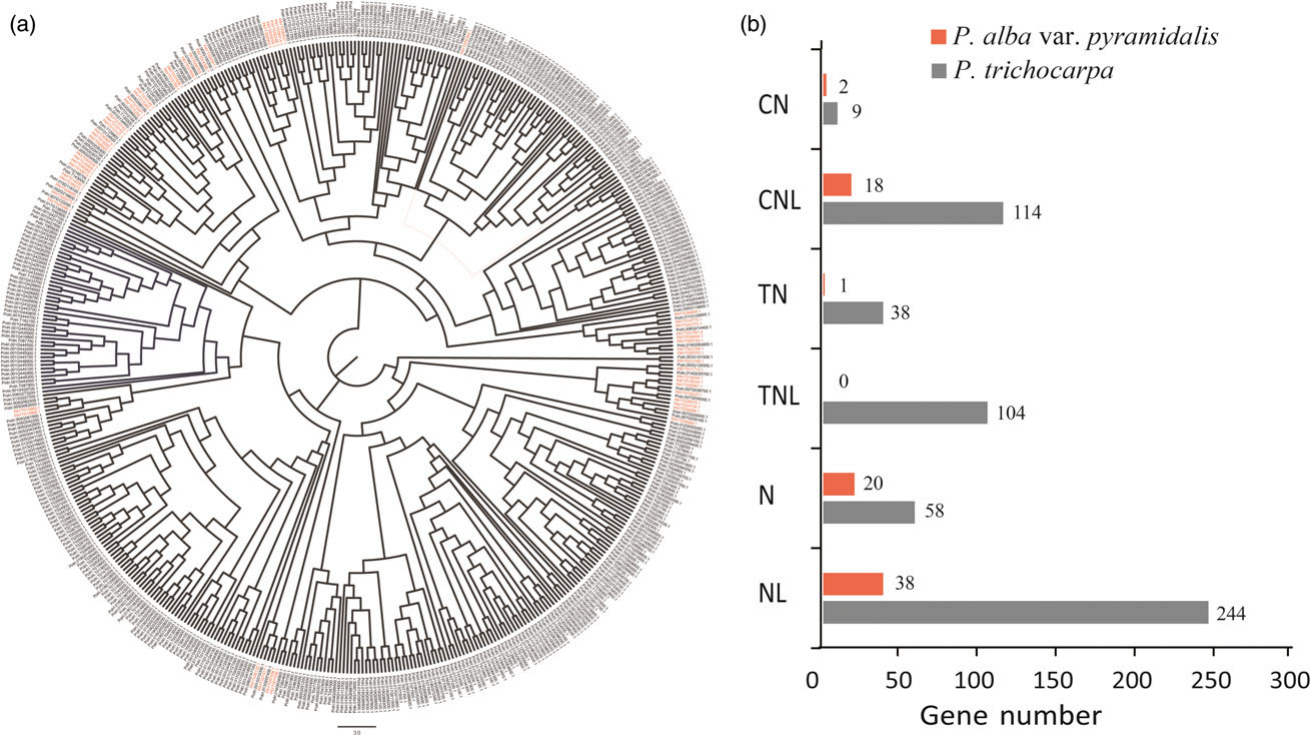
Phylogenetic tree of NBS genes and it's contraction in *Populus alba* var. *pyramidalis*. (a) Phylogenetic tree of NBS genes in *P. alba* var. *pyramidalis* and *P. trichocarpa*. (b) Numbers of NBS genes subfamilies in *P. alba* var. *pyramidalis* and *P. trichocarpa*.

In contrast, we found that 3234 gene families, including those containing nucleotide‐binding sites (NBSs) with key roles in plant disease resistance, were very much contracted in *P. alba* var. *pyramidalis* genome, with some genes containing the NBS domain being lost altogether (Figure 3; Table S11). For example, only one NBS gene copy containing TIR (Toll⁄interleukin‐1 receptor) domain, which belong to TN and TNL subfamilies, was found in *P. alba* var. *pyramidalis* genome (Figure 3b). Sequence alignment of these homologous genes showed that most TIR domains of NBS genes were lost in *P. alba* var. *pyramidalis* (Figure S4). The contraction of NBS gene family in *P. alba* var. *pyramidalis* genome was also confirmed when compared with other closely related Salicaceae species (Table S12).

### Genetic transformation efficiency and gene knock‐out in *P. alba* var. *pyramidalis*

**Figure 4:**
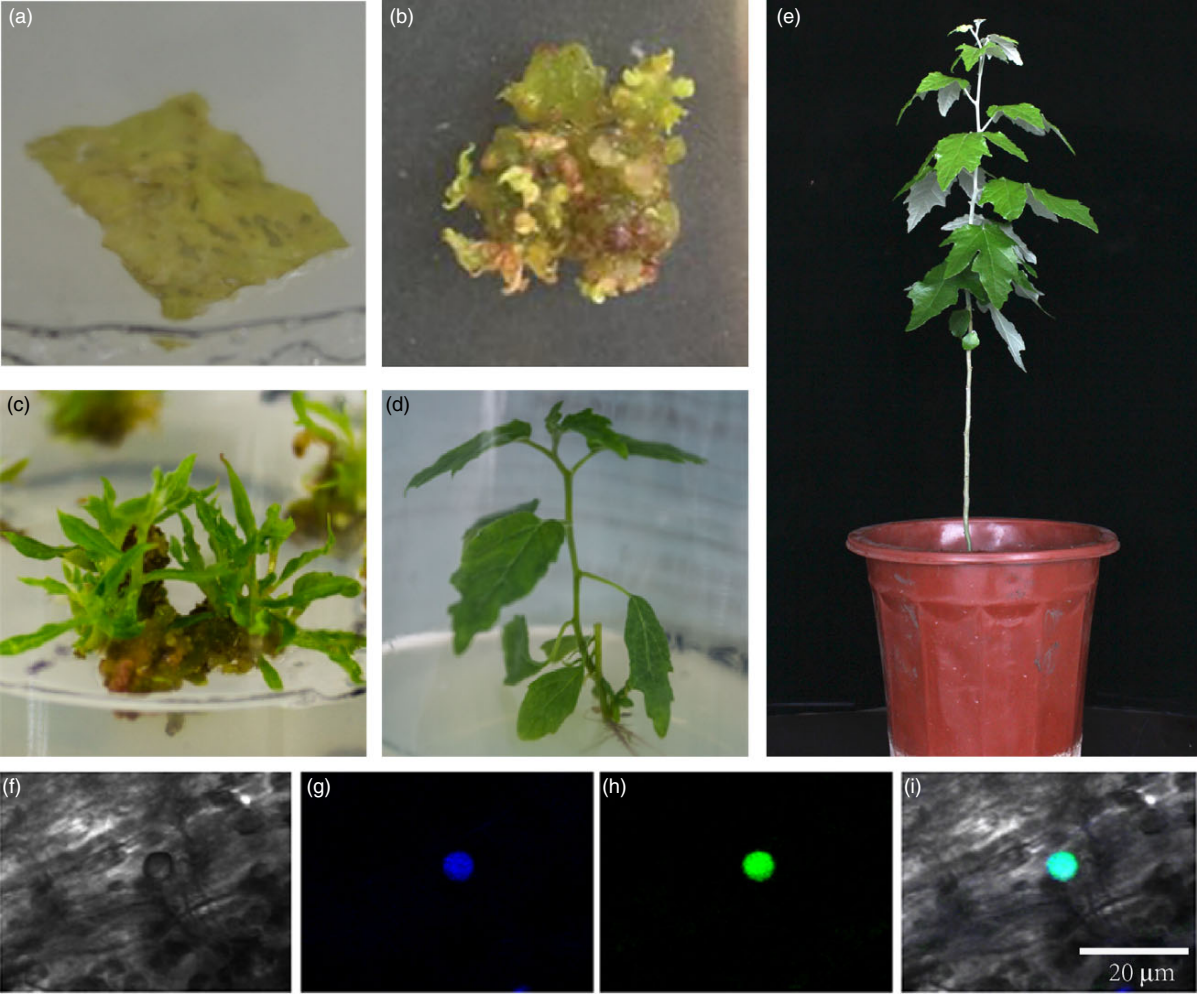
The genetic transformation process and results of transient transformation. (a) Simultaneous selection and regeneration on WPM‐reg plates. (b) Multiple green transgenic calli. (c) Transformed shoots regenerated from calli on WPM‐elo media. (d) A regenerated transgenic whole plant on rooting medium. (e) A transgenic plant under greenhouse conditions. (f–i) Transient GFP fusion protein expression assay in nucleus. (f) bright field; (g) DAPI; (h) green fluorescence; (i) overlay. Scale bar = 20 μm.

We next examined the efficiency of transformation of this variety with the standard *Agrobacterium*‐mediated system in poplars (Figure 4, Methods S1). We assessed different vectors for transferring the *Hyg* gene (Table 1). All young leaves subjected to co‐cultivation survived and we checked for the presence of the *Hyg* gene in at least one callus from each targeted leaf (Figure S5A). The average percentage of transgenic calli was about 80%. We then examined the success rate for inducing sprout regeneration and found that around 35.91% of the transgenic calli could produce shoots (Figure 4C,D). We excised these transgenic shoots from transgenic calli and cultured them in rooting medium. The average rooting efficiency was around 83.05%. The final transformation rates obtained were between 17.59% and 28.51% with an average rate of 23.6% (Table 1; Figure 4E). The average time from co‐cultivation to whole plant regeneration was about 80 days (Table S13).

**Table 1:** Summary of transformation efficiency results obtained with different vectors

Vector	Sprout induction (%)	Root induction (%)	Transgenesis rate (%)	Total efficiency (%)
pCAMBIA1305	34/94 (36.17)	77/85 (90.59)	67/77 (87.01)	28.51
PYL‐CRISPR‐CAS9‐HD	25/69 (36.23)	54/68 (79.41)	44/54 (81.48)	23.44
PYL‐CRISPR‐CAS9‐HD	25/73 (34.25)	25/37 (67.57)	19/25 (76.00)	17.59
PCXSN	19/56 (33.93)	68/77 (88.31)	36/50 (72.00)	21.57
PCXSN	24/70 (34.29)	19/24 (79.17)	17/19 (89.47)	24.29
pCAMBIA1302	38/101 (37.62)	95/106 (89.62)	33/50 (66.00)	22.25
pCAMBIA1302	35/90 (38.89)	78/90 (86.67)	41/50 (82.00)	27.64
Average	35.91	83.05	79.14	23.60

We also carried out a transient transformation assay for this variety, using a rapid transformation system for gene function analysis based on the method described by Takata and Eriksson (2012). We transferred a *C2H2*‐*AZF* gene in order to examine its intracellular localization in nucleus. Transient transformation was monitored by the expression of green fluorescence protein (GFP) from the vector (Figure 4F–I). Over 40% cells per leaf were found to show GFP signals in the nucleus under fluorescence stereomicroscope.

Finally, we performed gene knockout experiments in this variety. We followed the method of Fan *et al*. (2015) to knock out a *C2H2*‐*AZF* gene by means of a clustered regularly interspaced short palindromic repeats (CRISPR)‐associated protein (Cas) mediated system. We designed three guide RNAs to target the *C2H2*‐*AZF* gene and the knock‐out results were verified by performing qRT‐PCR and sequencing the PCR amplification products of the DNA fragments targeted. We found that the gene had been successfully knocked out in 89% (92 in total) of the samples (Figure S5B). Taken together, our experiments confirmed the high genetic transformation efficiency of *P. alba* (e.g. Soliman *et al*., 2017; Wang *et al*., 2008). Therefore, this species can be used for diverse molecular studies.

## Discussion

In this study, we reported the genome sequence of *P. alba* var. *pyramidalis* and examined its genomic differences among the closely related species. We found that this variety diverged from *P. trichocarpa* around 13 million years ago (Figure S1C). Both species had undergone two whole genome duplications and they exhibited extensive collinearity across the gene space (Figure 2B). We annotated 37 901 genes, similar to the total number of genes (41 335) identified in *P. trichocarpa*. At least 24 278 genes within the collinear regions between these two species are orthologous. We further identified 865 diversified gene pairs and 869 specie‐specific gene families, mainly enriched in abiotic stress response, will help *P. alba* var. *pyramidalis* adapted to diverse environment.

We also found a few genes to be species‐specific in *P. alba* var. *pyramidalis* due to the expansion of gene families involved in hormone metabolism and response. Hormones (especially auxin) are important factors affecting plant growth, and Aux/IAA proteins play a pivotal role in the perception and signalling of the hormone auxin (Liscum and Reed, 2002; Paponov *et al*., 2008). Importantly, we found that genes encoding protein containing the NBS domain were greatly contracted in *P. alba* var. *pyramidalis* compared with *P. trichocarpa*. These NBS genes play a critical role in disease resistance (including resistance to both bacteria and viruses) (Dangl and Jones, 2001). A total of 79 NBS gene copies were identified in *P. alba* var. *pyramidalis* genome, whereas 567, 251, 150, 419 and 205 copies were identified in *P. trichocarpa* (Tuskan *et al*., 2006), *P. euphratica* (Ma *et al*., 2013), *P. pruinosa* (Yang *et al*., 2017), *S. purpurea* (https://phytozome.jgi.doe.gov/pz/portal.html) and *S*. *suchowensis* (Dai *et al*., 2014), respectively (Table S12). These NBS genes were further classified into six subfamilies and all these subfamilies were contracted greatly in *P. alba* var. *pyramidalis*. It should be noted that gene copies and genomic structures vary not only greatly between species, but also between different genotypes of the same species according to the recent pan‐genome analyses (Pinosio *et al*., 2016; Zhang *et al*., 2018). However, how these genomic differences contribute to the species‐ or genotype‐specific traits need further studies for poplars in the future.

In addition, our experiments confirmed that the genetic transformation efficiency for *P. alba* is high as suggested before on this variety and the other genotype (Soliman *et al*., 2017; Wang *et al*., 2008). Therefore, this species may represent a useful new tree model for transformation‐based analyses for three reasons. First, leaves taken from cuttings can be used directly as material for transgenic experiments, which is preferable to the stem internodes used. Second, our final genetic transformation efficiency was on average 23.6%, a high value among poplars. Finally, we found that the entire process from co‐cultivation to whole plant regeneration required an average time of <3 months (80 days; Figure 4A–E), which could save a lot of time. Our subsequent transient transformation assays and gene knock‐out experiments similarly suggest it can be used for other molecular studies. In addition, some genotype of this species could start to flower far earlier than other poplars (Meilan *et al*., 2004) although it remains tested whether our transformation protocol works well for this genotype. All these findings indicate that *P. alba* shows high transformation efficiency and is likely to represent a new candidate model for genetic transformation and gene function tests in poplar tree species.

In conclusion, we reported the genome sequence of *P. alba* for the first time and confirmed its high transformation efficiency. Both the genome sequence and the transformation protocol presented here will accelerate our molecular understanding of this tree species, its breeding program and other diverse studies. Especially, we showed the genomic divergence between *P. alba* and other closely related species, which indicates that comparative genomic analyses through sequencing more species are necessary to a deep evolutionary understanding of the poplar adaption and diversification.

## Materials and methods

### Genome sequencing and assembly

Genomic DNA was extracted from leaf tissues of *P. alba* var. *pyramidalis* with a standard CTAB (cetyl trimethylammonium bromide) method (Porebski *et al*., 1997). We carried out whole genome shotgun sequencing with the Illumina Hiseq 2500 platform (Illumina, CA). Seven paired‐end sequencing libraries with insert sizes of approximately 270 bp, 500 bp, 800 bp, 2 kb, 5 kb, 10 kb and 20 kb were constructed, generating a total of 170 Gb of data. RNA samples were prepared from leaves, phloem, xylem, and roots of a 2‐year‐old individual and sequenced on the Illumina Hiseq 2500 platform (Illumina). 15 Gb of PacBio RS reads with an N50 of over 8 kb were sequenced on the PacBio RS II platform (Pacific Biosciences, CA).

We first generated the Illumina‐based *de novo* genome assembly using Platanus with *k*‐mer auto‐extension and the option “‐u = 0.2” (Kajitani *et al*., 2014). Next, all PacBio RS reads were used to fill the gaps by SSPACE‐LongRead v1‐1 (Boetzer *et al*., 2010) with default parameters after error correction by the Lordec software package v0.6 (Salmela and Rivals, 2014) with all the Hiseq 2500 short reads. Finally, PBJelly v15.8.24 (English *et al*., 2012) and GapCloser v1.12 (Li *et al*., 2008) were used with default parameters to improve the genome assembly.

### Repeat annotation

For transposable element annotation, RepeatMasker v4.05 (Tarailo‐Graovac and Chen, 2009) and RepeatProteinMasker (Tarailo‐Graovac and Chen, 2009) were used with default parameters against Repbase (Xu and Wang, 2007) to identify known repeats in the *P. alba* var. *pyramidalis* genome. In addition, RepeatModeler (Tarailo‐Graovac and Chen, 2009) and LTR_FINDER (Jurka *et al*., 2005) were used to identify *de novo* evolved repeats in the assembled genome. Parameters for LTR_FINDER were set to ‘Match = 2, Mismatch = 7, Delta = 7, PM = 80, PI = 10, Minscore = 50, and MaxPeriod = 2000’.

### Gene prediction and annotation

Three methods were used to predict protein‐coding genes: transcriptome‐based predictions, *de novo* predictions, and homology‐based predictions. For transcriptome‐based predictions, RNA from four tissues (leaves, xylem, phloem and root) was isolated and RNA‐seq data (NCBI SRR6003833–SRR6003836), processed by Trinity v2.2 (Grabherr *et al*., 2011), were used for gene annotation. For *de novo* predictions, Augustus v3.21 (Stanke *et al*., 2006), GenScan v1.4 (Burge and Karlin, 1997), glimmerHMM (Majoros *et al*., 2004), GeneMark v3.47 (Lukashin and Borodovsky, 1998) and SNAP (Korf, 2004) analyses were performed on the repeat‐masked genome, with parameters trained from transcriptome assembly data. Predicted protein sequences from *Arabidopsis thaliana*,* P. trichocarpa*,* Ricinus communis* and *Vitis vinifera* were used for homology‐based predictions with Phytozome v12 (Goodstein *et al*., 2011). The homology, *de novo* and transcriptomic gene sets were merged to form a comprehensive non‐redundant reference gene set using the EVidenceModeler (Haas *et al*., 2008) and PASA v2.0.2 (Haas *et al*., 2003) software packages. Functional annotation of the predicted gene models was based on comparison with the Swiss‐Prot (Boeckmann *et al*., 2003) and KEGG databases (Kanehisa and Goto, 2000) with a minimal e‐value of 1e‐5. GO terms were assigned to the annotated genes using the Blast2GO pipeline (version 3.1.3; Conesa *et al*., 2005). Protein domains and functions were analyzed using InterProScan (version 5.13–5. 20).

### Genome Quality Evaluation and gene clustering analyses

The qualities of the assembly and gene annotation were assessed using BUSCO v3 (Simão *et al*., 2015). We compared the *P. alba* var. *pyramidalis* genome sequence against a set of core eukaryotic genes using BUSCO. Syntenic blocks and gene collinearity were inferred using MCScanX (Wang *et al*., 2012) and Last software v2.28.2 (http://last.cbrc.jp/) and were visualized using Circos v0.69 (Krzywinski *et al*., 2009). Synonymous (*Ks*) and non‐synonymous (*Ka*) substitution rates for gene pairs were computed using the ‘YN00’ method from the PAML package v4.8 (Yang, 2007). To identify SNPs and indels in the *P. alba* var. *pyramidalis* genome, we mapped the sequenced short reads to the draft *P. alba* var. *pyramidalis* genome using BWA v0.7.12‐r1039 (Li and Durbin, 2009) and called SNPs and indels using Samtools v0.1.19‐44428 cd (Li *et al*., 2009).

Ortholog clustering analysis was performed using OrthoMCL v2.0.9 (Li *et al*., 2003) applied to all the protein‐coding genes of *P. alba* var. *pyramidalis* and *A. thaliana*,* Manihot esculenta*,* Linum usitatissimum*,* Salix purpurea*,* V. vinifera*,* Oryza sativa*,* Carica papaya*,* P. trichocarpa*,* P. euphratica* and *R. communis*. The MCMCTREE program, implemented in the PAML package v4.8 (Yang, 2007), was used to estimate divergence times with calibration times referred to Ma *et al*. (2013). The phylogenetic tree was constructed from single copy genes by PhyML (Guindon *et al*., 2010). In order to compare variations of gene copies between *P. alba* and closely related species, we further downloaded genomes of *P. euphratica* (Ma *et al*., 2013), *P. pruinosa* (Yang *et al*., 2017), *S. purpurea* (https://phytozome.jgi.doe.gov/pz/portal.html), *S*. *suchowensis* (Dai *et al*., 2014) and *P. trichocarpa* (Tuskan *et al*., 2006). Although genomes of *P. tremula* and *tremuloides* are also available through PopGenIE (http://popgenie.org/), the poor assemblies limit their comparisons with *P. alba* and other species. Species‐specific gene families were identified with the cluster of genes form only one specie. Gene expansion and contraction analysis was conducted using the CAFÉ program (version 3.1; De Bie *et al*., 2006) with information from the estimated phylogenetic tree. The Hidden Markov Model (Eddy, 1998) profile for domains from the Pfam database (26.0; Finn *et al*., 2009) and HMMER software (version 3.1; Finn *et al*., 2011) were used to identify gene families. Resistance genes were identified by the presence of the NBS domain and classified into six groups (CN: CC‐NBS; CNL: CC‐NBS‐LRR; TN: TIR‐NBS; TNL: TIR‐NBS‐LRR; N: NBS; NL: NBS‐LRR).

### Genetic transformation process

One‐year‐old *P. alba* var. *pyramidalis* clones propagated from cuttings grown in a greenhouse at 25 °C under cycles of 16 h light/8 h darkness (6:30–22:30; 100 μmol/m^2^/s) and 60% humidity, were used for transformation. After disinfecting with 12% sodium hypochlorite, the leaves of *P. alba* var. *pyramidalis* was cut into pieces and put on Woody Plant Medium (with 2 mg/L zeatin, 1 mg/L naphthalene acetic acid and 100 μmol/L acetosyringone) for induction. When the explants had been induced to produce new plants under aseptic conditions, they could be used for the transformation process. This was performed according to the *P. alba* var. *pyramidalis* transformation protocol given in Methods S1. The transformation time and success rate were calculated for each step.

### Transient transformation process

Sterile rooted cuttings from *P. alba* var. *pyramidalis* clones, grown in a greenhouse at 25 °C under cycles of 16 h light/8 h darkness (100 μmol/m^2^/s), were used for transient transformation. The pCXDG‐based expression vector employed here harbours a GFP gene driven by the Cauliflower Mosaic Virus 35S (CaMV35S) promoter. The expression vectors were transformed into *Agrobacterium tumefaciens* strain GV3101. *Agrobacterium* harbouring individual vectors was inoculated into YEP media with appropriate antibiotics. An overnight culture of *Agrobacterium* was harvested at an OD_600_ of 0.3, centrifuged at 5000 × g for 10 min, and re‐suspended in 50 mL of infiltration medium (0.5 × MS medium containing 5 mm MES‐KOH (pH 5.6), and 200 μM acetosyringone) to an OD_600_ of 0.3. The bacterial suspension was incubated at room temperature for three hours with gentle shaking in the dark. Then *Agrobacterium* infiltration was performed by applying a vacuum three time for three minutes. The cuttings were then put on paper towels to remove excess infiltration medium and transplanted into 0.5 × MS medium (pH 5.6) with 0.6% (w/v) agar and 50 μg/mL cefotaxime. We followed the method of Takata and Eriksson (2012) to conduct a transient transformation assay of the *C2H2*‐*AZF* (PAYT023741.1) gene in order to measure expression in the nucleus. The cuttings were returned to the initial growing conditions for 3 days before imaging. Images of whole leaves were monitored using a fluorescence stereomicroscope (Leica TCS SP8, Germany) with excitation at 488 nm to detect GFP fluorescence.

### Gene knock‐out experiments

We followed the procedures of Fan *et al*. (2015) to perform gene knock‐out experiments. One‐year‐old *P. alba* var. *pyramidalis* clones propagated from cuttings were used for CRISPR/Cas9‐mediated targeted mutagenesis. The AZF genomic DNA fragment was amplified by PCR with gene‐specific primers (AZF‐F: 5′‐ACCTTTCCTTCTCTCTTCGGAT‐3′; AZF‐R: 5′‐TCCAACAATCTTCCTAATTGAACCT ‐3′). The PCR product was cloned and sequenced, and the sequence was used to select CRISPR/Cas9 target sites. Three output target sites were selected for designing sgRNA sequences based on their locations in the gene and their GC contents. Three target sites were assembled in plasmids designated ATU3b, ATU6‐1 and ATU6‐29 with the specific primers (ATU3b‐F: 5′‐gtcaTCGTAGTGATTCCCCTTCAA‐3′, ATU3b‐R: 5′‐aaacTTGAAGGGGAATCACTACGA‐3′; ATU6‐1F:5′‐ attgTTGAAAGGAGTGGCTGTTGT‐3′, ATU6‐1R: 5′‐ aaacACAACAGCCACTCCTTTCAA‐3′; ATU6‐29F: 5′‐attgCGCCACGAGCGAGCATGATA‐3′, ATU6‐29R: 5′‐ aaacTATCATGCTCGCTCGTGGCG‐3′). The binary pYLCRISPR/Cas9 multiplex genome targeting vector system carrying the CAS9 coding gene and three plasmids with sgRNA cassettes driven by AtU3b, AtU6‐1 and AtU6‐29 were generated. After completion of the transgenic procedure, the plants obtained were selected on 9 mg/L hygromycin and the mutations they contained were identified through Sanger sequencing of individual clones; the mutation rate in transgenic plants was calculated according to the ratio of mutated clonal amplicons to total sequenced clonal amplicons.

### Availability

Sequence data from this study can be found at the National Center for Biotechnology Information website (http://www.ncbi.nlm.nih.gov) under SRA accession number SRR5990011 (library with 10 kb insert size), SRR5990012 (20 kb), SRR5990014 (270 bp), SRR5990015 (2 kb), SRR5990016 (500 bp), SRR5990017 (5 kb), SRR5990018 (800 bp) and SRR5990031 (PacBio RS data). The clone sources of *P. alba* var. *pyramidalis* can be obtained through the corresponding author. The whole genome sequence data and the annotation file reported in this paper have been deposited in the Genome Warehouse in BIG Data Center (BIG Data Center Members, 2018), Beijing Institute of Genomics (BIG), Chinese Academy of Sciences, under accession number GWHAAEP00000000 that is publicly accessible at http://bigd.big.ac.cn/gwh.

## Funding

The research was supported by the National Key Research and Development Program of China (2016YFD0600101), the National High‐Tech Research and Development Program of China (2013AA102605), the National Science Foundation of China (31561123001, 31470620 and 31500502) and the ‘111’ collaboration project.

## Conflict of interest

The authors declare no conflict of interest.

## Supporting information


**Methods S1** The protocol of *Populus alba* var. *pyramidalis* genetic transformation system.
**Figure S1** Genome assembly process and characteristics.
**Figure S2** GO enrichment of specie‐specific genes.
**Figure S3** Phylogenetic tree and expression conditions of PUP gene family and Aux/IAA proteins.
**Figure S4** Homologous NBS R genes in *Populus alba* var. *pyramidalis* were lost TIR and NBS domain.
**Figure S5** Transformation of *Populus alba* var. *pyramidalis*.
**Table S1** Sequencing data used for genome assembly.
**Table S2** Assemble features of *Populus alba* var. *pyramidalis* genome.
**Table S3** Summary of transposon content in the genome.
**Table S4** Summary of predicted protein‐coding gene annotations and their supporting evidence types.
**Table S5** RNA‐seq data of four tissues.
**Table S6** Functional annotation of predicted genes.
**Table S7** Evaluation of completeness of the genome assembly using BUSCOs.
**Table S8** Annotation of non‐coding RNAs.
**Table S9** GO enrichment of high diversification gene pairs.
**Table S10** GO enrichment of expanded genes families.
**Table S11** GO enrichment of contracted genes families.
**Table S12** NBS gene numbers identified in different poplars.
**Table S13** Summary of culturing time of different transformation stage.
