# Supplementary material for: Genome sequence and genetic transformation of a widely distributed and cultivated poplar
Source: Plant Biotechnol J. 2018 Oct 24;17(2):451–60. doi: 10.1111/pbi.12989 (PMC6335071; doi:10.1111/pbi.12989)
Supplement: Supplementary file 1 — Methods S1 The protocol of Populus alba var. pyramidalis genetic transformation system. Figure S1 Genome assembly process and characteristics. Figure S2 GO enrichment of specie‐specific genes. Figure S3 Phylogenetic tree and expression conditions of PUP gene family and Aux/IAA proteins. Figure S4 Homologous NBS R genes in Populus alba var. pyramidalis were lost TIR and NBS domain. Figure S5 Transformation of Populus alba var. pyramidalis. Table S1 Sequencing data used for genome assembly. Table S2 Assemble features of Populus alba var. pyramidalis genome. Table S3 Summary of transposon content in the genome. Table S4 Summary of predicted protein‐coding gene annotations and their supporting evidence types. Table S5 RNA‐seq data of four tissues. Table S6 Functional annotation of predicted genes. Table S7 Evaluation of completeness of the genome assembly using BUSCOs. Table S8 Annotation of non‐coding RNAs. Table S9 GO enrichment of high diversification gene pairs. Table S10 GO enrichment of expanded genes families. Table S11 GO enrichment of contracted genes families. Table S12 NBS gene numbers identified in different poplars. Table S13 Summary of culturing time of different transformation stage. [file PBI-17-451-s001.docx]

**Genome sequence and genetic transformation of a widely distributed and cultivated poplar**

Jianchao Ma^1†^, Dongshi Wan^1†^, Bingbing Duan^1^, Xiaotao Bai^1^, Qiuxian Bai^1^, Ningning Chen^1^, Tao Ma^1,2*^

^1^State Key Laboratory of Grassland Agro-Ecosystem, School of Life Sciences, Lanzhou University, Lanzhou 730000, China;

^2^Key Laboratory of Bio-Resource and Eco-Environment of Ministry of Education, College of Life Sciences, Sichuan University, Chengdu 610065, China

^†^ These authors contributed equally to this work.

*Corresponding author. E-mail: matao.yz@gmail.com

**Supporting information legends**

**Methods S1.** The protocol of *P. alba* var. *pyramidalis* genetic transformation system.

**Figure S1.** Genome assembly process and characteristics.

**Figure S2.** GO enrichment of specie-specific genes.

**Figure S3.** Phylogenetic tree and expression conditions of PUP gene family and Aux/IAA proteins.

**Figure S4.** Homologous NBS R genes in *P. alba* var. *pyramidalis* were lost TIR and NBS domain.

**Figure S5.** Transformation of *P. alba* var. *pyramidalis*.

**Table S1.** Sequencing data used for genome assembly.

**Table S2.** Assemble features of *P. alba* var. *pyramidalis* genome.

**Table S3.** Summary of transposon content in the genome.

**Table S4.** Summary of predicted protein-coding gene annotations and their supporting evidence types.

**Table S5.** RNA-seq data of four tissues.

**Table S6.** Functional annotation of predicted genes.

**Table S7.** Evaluation of completeness of the genome assembly using BUSCOs.

**Table S8.** Annotation of non-coding RNAs.

**Table S9.** GO enrichment of high diversification gene pairs.

**Table S10.** GO enrichment of expanded genes families.

**Table S11.** GO enrichment of contracted genes families.

**Table S12.** NBS gene numbers identified in different poplars.

**Table S13.** Summary of culturing time of different transformation stage.

**Supplementary Methods 1: The protocol of *P. alba* var. *pyramidalis* genetic transformation system**

**Activation of agrobacterium**

1) The agrobacterium strain containing recombinant plasmids was streaked onto YEP solid medium (50 mg/L Kan + 40 mg/L Rif) and cultured at 28 ℃ for 40~48 hours;

2) Inoculate 25 mL YEP liquid medium containing 50 mg/L Kan and 40 mg/L Rif with a single clone and grow at 28℃ with shaking until OD_600_ is 0.6~0.8;

3) Inoculate 25 mL fresh YEP medium added antibiotics with 200 μL culture from last step and grow at 28℃ with shaking until OD_600_ is 0.3~0.5;

4) Take all culture and centrifuge at 4000 rpm, 4 ℃, for 10 min;

5) Resuspend the pellet in 30 ml WPM suspension medium and shake the suspension at 28℃ for 1~2 h prepared for transformation.

**Agrobacterium-mediated genetic transformation**

For transformation, young leaves of aseptic seedlings were selected and cut into 0.5 x 0.5 cm^2^ discs, then dipped in bacterial suspension for 10 min (shake lightly the suspension every 2~3 min).

1. **Incubation**

Move the dipped leaf discs onto dry sterilized papers to remove the liquid and place them onto WPM co-culture solid medium (abaxial leaf surface down) and incubate these materials in dark for 2 days at 25℃.

1. **Callus inducing**

After 2 days of incubation with agrobacterium, the leaf discs are transferred to WPM selection medium and incubated at 25℃ in dark for 18~28 days (replaced medium every 10 days) until white loose callus appeared around the leaf discs.

1. **Sprout inducing**

Move the callus onto the WPM shooting medium to induce sprouts. The parameter setting of incubator is temperature: 25 ℃; light: 2000~10000 Lux; dark/day cycle: 8 h/16 h for about 30~40 days (replace the medium every 10 days) until adventitious buds appears.

1. **Root inducing**

Cut and move the buds which are approximately 3~4 cm into WPM rooting medium until roots appeared.

1. **Transplantation of transgenic *P. alba* var. *pyramidalis***

Remove agar from the roots of seedlings and transplant the entire *P. alba* into soil, then cultivate these plants in greenhouse.

**Mediums:**

WPM basic medium (subculture): WPM + 30 g/L sucrose + 8.0~8.1 g/L agar + 0.05 mg/L NAA;

WPM suspension medium: WPM + 30 g/L sucrose + 100 μmol/L AS;

WPM co-culture medium: WPM+30 g/L sucrose +100 mol/L AS + 1mg/L NAA + 100mg/L ZT + 7.6~7.8 g/L agar;

WPM selection medium: WPM + 30 g/L sucrose +90mg/L Hyg + 1 mg/L NAA +100 mg/L ZT + 400 mg/L Cef + 7.6~7.8 g/L agar;

WPM shooting medium: WPM + 30 g/L sucrose +90 mg/L Hyg +0.1 mg/L NAA +100 mg/L ZT + 400 mg/L Cef + 7.4~7.6 g/L agar;

WPM rooting medium: WPM + 30 g/L sucrose + 90 mg/L Hyg + 0.1 mg/L NAA + 400 mg/L Cef + 8.0~8.1 g/L agar;

All mediums are needed to adjust pH value to 5.80~5.85 before adding agar and then sterilized at 121℃ for 20 min. All antibiotics and ZT are needed to be added after sterilization. The WPM medium could be replaced by MS medium.


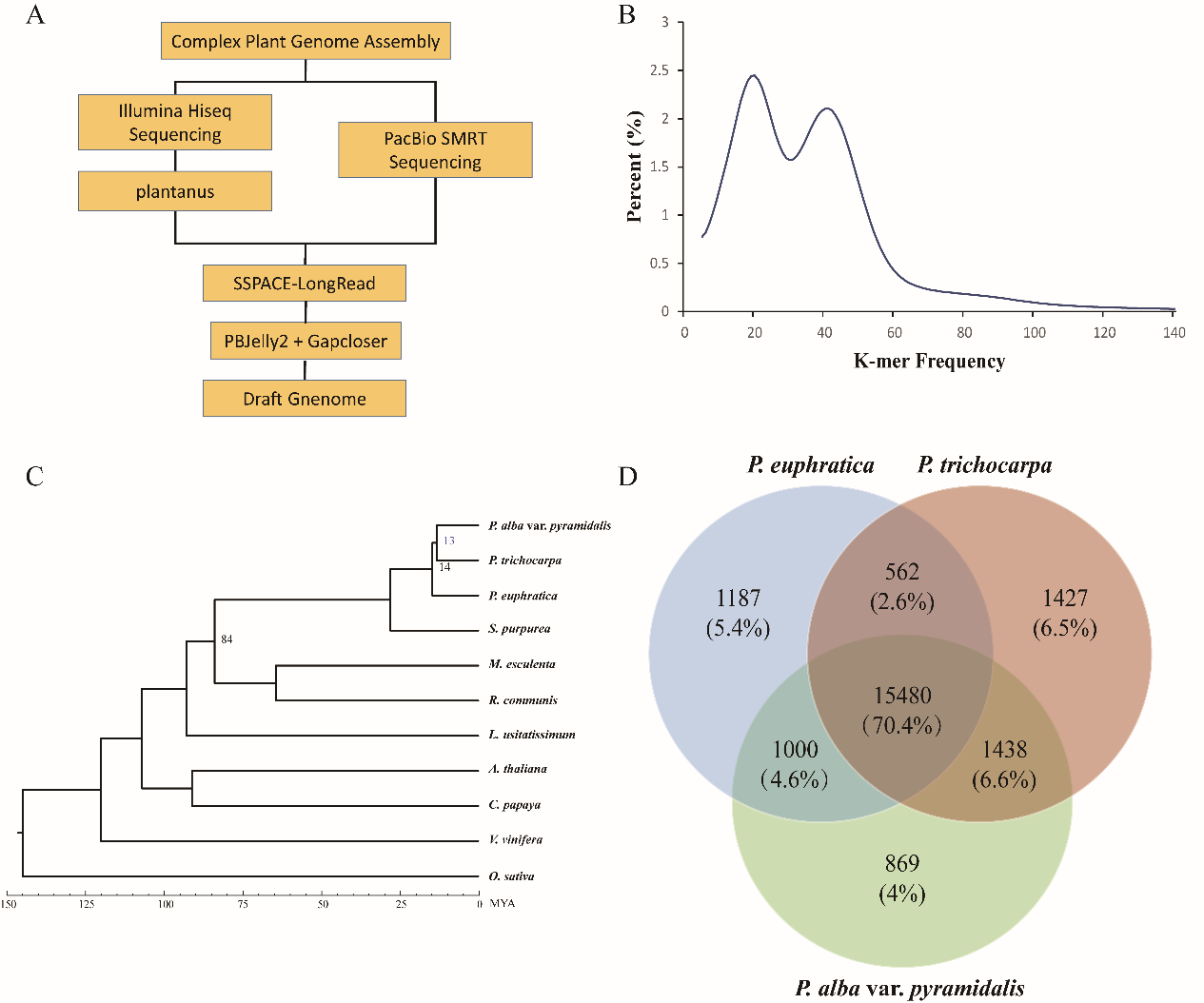


**Supplementary Figure 1: Genome assembly process and characteristics.**

(A) Flowchart of the approaches used for de novo assembly.

(B) Distribution of sequence depth across the assembled genome. The Y-axis represents the proportion of the genome at a given sequencing depth.

(C) The divergence times estimated by MCMCTree.

(D) Venn diagram comparing the number of gene families shared between *P. alba*var. *pyramidalis*, *P. euphratica* and *P. trichocarpa*.


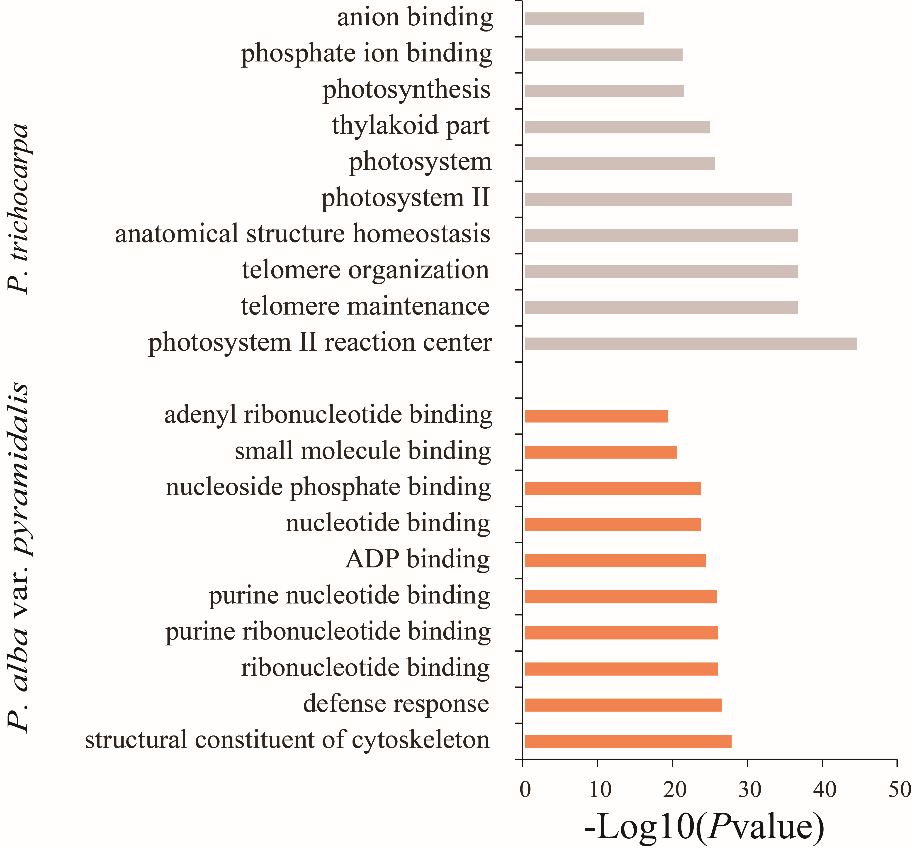


**Supplementary Figure 2: GO enrichment of specie-specific genes.**


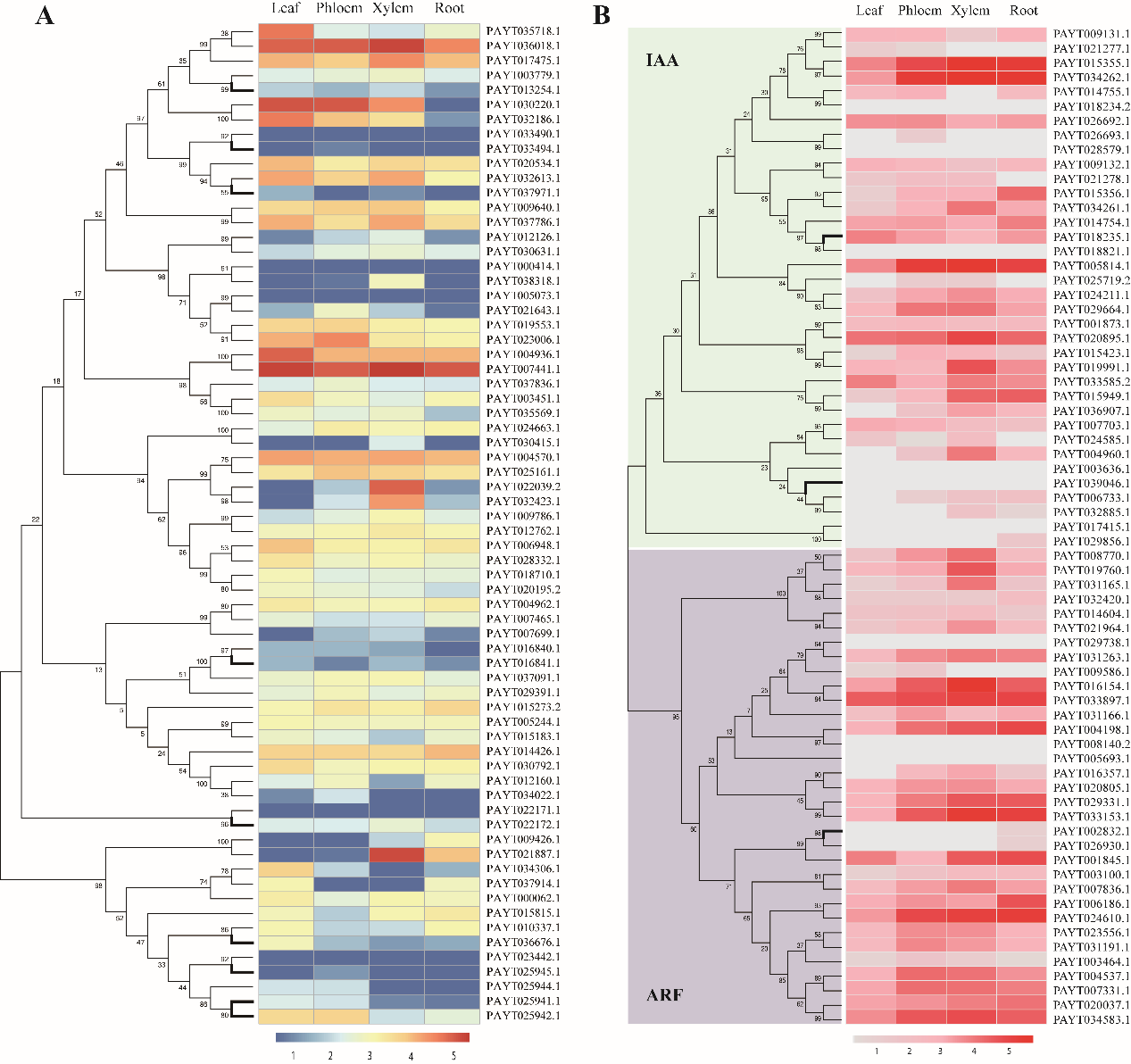


**Supplementary Figure 3: Phylogenetic tree and expression conditions of PUP gene family and Aux/IAA proteins.**

1. Phylogenetic tree and expression conditions of PUP gene family. The bold line indication expanded genes in *P. alba*var. *pyramidalis* compared with *P. trichocarpa*.
2. Phylogenetic tree and expression conditions of Aux/IAA proteins. The bold line indication expanded genes in *P. alba*var. *pyramidalis* compared with *P. trichocarpa*.

**Supplementary Figure 4: Homologous NBS R genes in *P. alba*var. *pyramidalis* were lost NBS domain.**

**
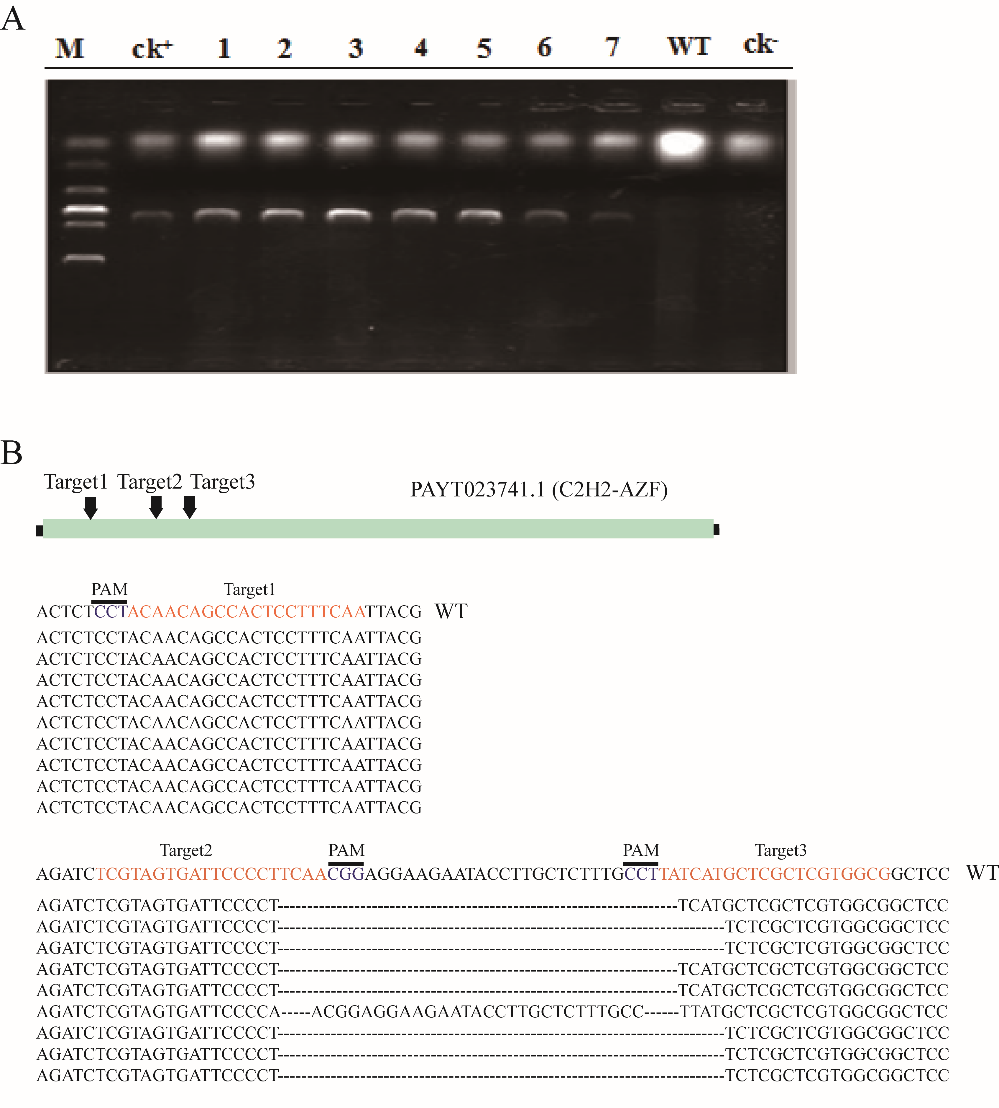
**

**Supplementary Figure 5: Transformation of *P. alba* var. *pyramidalis*.**

1. Transformation verification. Only 7 clones were presented. ‘ck+’ and ‘ck-’ means positive and negative control. The size of PCR product is about 750 bp.
2. CRISPR/Cas9-mediated target sequences.

**Supplementary Table 1: Sequencing data used for genome assembly.**

| Platpform | Library(bp) | Average read length (bp) | Read count | Read base(G) | Sequence depth |
| --- | --- | --- | --- | --- | --- |
| Illumina | 270 | 150 | 308,046,536 | 46.21 | 87.18 |
|  | 500 | 125 | 363,356,698 | 45.42 | 85.70 |
|  | 800 | 125 | 131,050,720 | 16.38 | 30.91 |
|  | 2000 | 49 | 486,094,260 | 23.82 | 44.94 |
|  | 5000 | 49 | 213,744,640 | 10.47 | 19.76 |
|  | 10000 | 49 | 236,211,174 | 11.57 | 21.84 |
|  | 20000 | 49 | 328,347,286 | 16.09 | 30.36 |
|  | total |  | 279,450,919 | 169.96 | 320.69 |
| PacBio |  | 3954 | 3,988,040 | 15.76 | 29.74 |

**Supplementary Table 2: Assemble feathures of *P. alba* var. *pyramidalis* genome.**

| Genome assembly | Illunima Data | Illumina + Pacbio Data |
| --- | --- | --- |
| No. Scaffolds | 42789 | 17797 |
| Total span | 406769449 bp | 464516523 bp |
| Average scaffold length | 9506 bp | 26100 bp |
| N50, scaffold | 348883 bp | 459178 bp |
| N90, scaffold | 3717 bp | 10019 bp |
| Longest scaffold | 2284541 bp | 3427229 bp |
| No. contigs | 95336 | 55988 |
| Longest contigs | 85678 bp | 230279 bp |
| N50, contig | 9767 bp | 26535 bp |
| N90, contig | 1496 bp | 3880 bp |
| GC content | 33.50% | 33.70% |
| N percent in genome | 8.50% | 3% |

**Supplementary Table 3: Summary of transposon content in the genome.**

| Type | *Populus alba* var. *pyramidalis* | | *P. euphratica* | | *P. trichocarpa* | |
| --- | --- | --- | --- | --- | --- | --- |
|  | Length (bp) | % of genome | Length (bp) | % of genome | Length (bp) | % of genome |
| DNA | 29,051,312 | 6.45 | 15,607,047 | 3.14 | 49,746,165 | 11.46 |
| LINE | 6,323,112 | 1.40 | 6,259,522 | 1.26 | 9,517,483 | 2.19 |
| LTR | 92,114,532 | 20.45 | 182,167,329 | 36.69 | 143,464,168 | 33.05 |
| SINE | 2,279,639 | 0.51 | 998,670 | 0.2 | 31,815 | 0.01 |
| Simple Repeat | 5,711,330 | 1.27 | 1,496,844 | 0.3 | 999,522 | 0.23 |
| Unknown | 46,575,303 | 10.34 | 35,600,191 | 7.17 | 7,659,748 | 1.76 |
| Total | 200,969,633 | 44.61 | 212,422,255 | 42.78 | 201,114,633 | 46.33 |

**Supplementary Table 4: Summary of predicted protein-coding gene annotations and their supporting evidence types.**

| Gene set | | Total Genes Predicted | Average Gene Length (bp) | Average CDS Length (bp) | Average Exons per Gene | Average Exon Length (bp) | Average Intron Length (bp) |
| --- | --- | --- | --- | --- | --- | --- | --- |
| *De novo* | AUFUSTUS | 31434 | 3152.90 | 1258.95 | 5.52 | 227.87 | 415.51 |
|  | GENEMARK | 42010 | 3259.00 | 1108.19 | 5.18 | 213.97 | 514.65 |
|  | GENEID | 45519 | 4757.08 | 915.02 | 4.70 | 194.61 | 1037.87 |
|  | SNAP | 47719 | 2564.16 | 909.63 | 4.82 | 188.62 | 432.84 |
|  | GLIMMERHMM | 37716 | 2191.12 | 966.89 | 4.04 | 239.11 | 402.22 |
| Homolog | *A. thaliana* | 27400 | 2525.24 | 1102.65 | 4.72 | 233.77 | 395.72 |
|  | *P. euphratica* | 34466 | 2571.66 | 1106.52 | 4.60 | 240.44 | 420.08 |
|  | *P. trichocarpa* | 39036 | 2269.82 | 1025.10 | 4.33 | 236.86 | 386.01 |
|  | *R. communis* | 31499 | 2427.69 | 1075.01 | 4.47 | 240.44 | 402.76 |
|  | *V. vinifera* | 29576 | 2502.05 | 1039.79 | 4.84 | 214.83 | 392.48 |
| RNA_seq | | 29121 | 3119.30 | 833.09 | 3.57 | 233.59 | 464.61 |
| EVM | | 39238 | 2754.64 | 1095.89 | 4.85 | 226.00 | 430.95 |
| Final set | | 37901 | 3230.95 | 1102.87 | 4.80 | 229.64 | 432.46 |

**Supplementary Table 5: RNA-seq data of four tissues.**

| Samples | length | TotalNumber | MappedReads | TotalBase(Gb) |
| --- | --- | --- | --- | --- |
| Leaf | 125 | 44460642 | 66158366 | 11.12 |
| Phloem | 125 | 40681278 | 61157886 | 10.17 |
| Xylem | 125 | 44738497 | 71921563 | 11.18 |
| Root | 125 | 47242542 | 78743715 | 11.81 |

**Supplementary Table 6: Functional annotation of predicted genes.**

| **Database** | **Number** | **Percent (%)** |
| --- | --- | --- |
| Total | 37901 |  |
| InterPro | 28944 | 76.4 |
| GO | 28707 | 75.7 |
| KEGG | 10511 | 27.7 |
| Swissprot | 27744 | 73.2 |
| Annotated | 32513 | 85.8 |
| Unannotated | 5388 | 14.2 |

**Supplementary Table 7: Evaluation of completeness of the genome assembly using BUSCOs.**

| **Species** | **BUSCO Terms** | **Genome** | **Protein** |
| --- | --- | --- | --- |
| *Populus alba* var. *pyramidalis* | % Complete BUSCOs | 91.10% | 97.00% |
|  | % Fragmented BUSCOs | 2.00% | 1.70% |
|  | % Missing BUSCOs | 6.90% | 1.30% |
| *P. euphratica* | % Complete BUSCOs | 90.10% | 92.40% |
|  | % Fragmented BUSCOs | 2.00% | 4.30% |
|  | % Missing BUSCOs | 7.90% | 3.30% |
| *P. trichocarpa* | % Complete BUSCOs | 92.10% | 98.70% |
|  | % Fragmented BUSCOs | 1.00% | 0.70% |
|  | % Missing BUSCOs | 6.90% | 0.60% |

**Supplementary Table 12:** **NBS gene numbers identified in different poplars.**

| Type | *P. alba* var. *pyramidalis* | *P. trichocarpa* | *P. euphratica* | *P. pruinosa* | *S. purpurea* | *S. suchowensis* |
| --- | --- | --- | --- | --- | --- | --- |
| CN | 2 | 9 | 6 | 3 | 2 | 10 |
| CNL | 18 | 114 | 43 | 30 | 101 | 55 |
| TN | 1 | 38 | 6 | 3 | 3 | 1 |
| TNL | 0 | 104 | 51 | 5 | 91 | 4 |
| N | 20 | 58 | 28 | 31 | 15 | 19 |
| NL | 38 | 244 | 117 | 78 | 207 | 116 |
| Total | 79 | 567 | 251 | 150 | 419 | 205 |

**Supplementary Table 13: Summary of culturing time of different transformation stage.**

| Lines | Callus inducing(day) | Sprout inducing(day) | Root inducing(day) | Total time(day) |
| --- | --- | --- | --- | --- |
| 1 | 21 | 43 | 9 | 73 |
| 2 | 25 | 50 | 7 | 82 |
| 3 | 23 | 51 | 8 | 82 |
| 4 | 19 | 44 | 10 | 73 |
| 5 | 27 | 53 | 8 | 88 |
| 6 | 25 | 50 | 9 | 84 |
| Average | 23 | 48.5 | 8.5 | 80 |
